# A new R package to parse plant species occurrence records into unique collection events efficiently reduces data redundancy

**DOI:** 10.1038/s41598-024-56158-3

**Published:** 2024-03-05

**Authors:** Pablo Hendrigo Alves de Melo, Nadia Bystriakova, Eve Lucas, Alexandre K. Monro

**Affiliations:** 1 IFMG - Instituto Federal de Educação, Ciência e Tecnologia de Minas Gerais, Campus Avançado Piumhi, Rua Severo Veloso, 1880 - Bairro Bela Vista, Piumhi, Minas Gerais 37925-000 Brazil; 2 The Natural History Museum, Cromwell Road, London, SW7 5BD UK; 3 Royal Botanic Gardens, Kew, Richmond, London, TW9 3AE UK

**Keywords:** Digital duplicates, Gatherings, Global biodiversity information facility, Myrtaceae, Species taxonomic names, Synonyms, Taxonomic inflation, World checklist of vascular plants, Classification and taxonomy, Computational platforms and environments, Data acquisition, Data integration, Data mining, Data processing, Databases, Software

## Abstract

Biodiversity data aggregators, such as Global Biodiversity Information Facility (GBIF) suffer from inflation of the number of occurrence records when data from different databases are merged but not fully reconciled. The ParseGBIF workflow is designed to parse duplicate GBIF species occurrence records into unique collection events (gatherings) and to optimise the quality of the spatial data associated with them. ParseGBIF provides tools to verify and standardize species scientific names according to the World Checklist of Vascular Plants taxonomic backbone, and to parse duplicate records into unique ‘collection events’, in the process compiling the most informative spatial data, where more than one duplicate is available, and providing crude estimates of taxonomic and spatial data quality. When GBIF occurrence records for a medium-sized vascular plant family, the Myrtaceae, were processed by ParseGBIF, the average number of records useful for spatial analysis increased by 180%. ParseGBIF could therefore be valuable in the evaluation of species’ occurrences at the national scale in support for national biodiversity plans, identification of plant areas important for biodiversity, sample bias estimation to inform future sampling efforts, and to forecast species range shifts in response to global climate change.

## Introduction

We are in a biodiversity and climate crisis in which almost 40% of plant species are threatened^1^. A confounding factor for our attempts to mitigate the impacts of Climate Change and the Biodiversity Crisis is that we know relatively little about plant occurrences^2^, and much of what we think that we do know is based on species distribution models.

Global plans to mitigate the biodiversity crisis include the High Ambition Coalition 30 × 30 initiative, a commitment to conserve 30% of the terrestrial environment by 2030, and the Global Biodiversity Framework (GBF) of the Convention on Biological Diversity (CBD). Both require adequate knowledge of species and their occurrences to be effective^3^. Such knowledge is, however, lacking for most of the world’s biodiversity hotspots, with much of the tropics being characterised by low numbers of observations and very biased sampling^4,5^. For example, in GBIF the plant diversity of the Amazon forest biodiversity hotspot is documented by an average of 10 to 14 (Source: GBIF, biological collections occurrence data) occurrence records per 100 km^2^. In contrast, the Mesoamerican biodiversity hotspot in Costa Rica is documented by 10,539 records per 100 km^2^ (Source: GBIF, biological collections occurrence data). Such gaps can be the result of a lack of primary data (sample effort), or of primary data not having been digitised, and or, incorporated into GBIF (data capture). Our knowledge of plant diversity is therefore both very uneven and incomplete, making it unsuitable for managing hotspots for plant conservation at this critical time. As a result, much of our knowledge on species richness and distribution is inferred using distribution models and interpolation, both of which are problematic if not validated or when used to identify hotspots of diversity and priority areas for conservation^6^. More recent applications of machine learning to address this problem include those by Richard-Bollans et al.^7^ and Cai et al.^8^.

The Global Biodiversity Information Facility (GBIF) is the largest occurrence data aggregator, with over 2.3 billion records (accessed June 2023). With the aggregation of occurrences records, new challenges emerge, including inconsistencies in backbone taxonomy across databases and large numbers of duplicate records. This phenomenon arises because of several physical duplicates of biological specimens being collected at the same time, which are then distributed to several institutions, and in the process, accumulate conflicting species identities and localities. Sources of conflict can include differences in taxonomic opinion/competence, approaches to transcribing and uploading of the data to information facilities. In addition, as identified by Feng et al.^9^, “some databases develop backbone systems (e.g., BIE backbone, GBIF backbone, MOL backbone), some databases adopt a name scrubbing tool that standardizes names towards pre-selected taxonomic systems (e.g., BIEN, GIFT, TRY), some rely on multiple taxonomic systems (e.g., iNaturalist, EOL), and some do not implement a strong regulation on taxonomic names (e.g., VertNet)”. The above leads to the inflation of the number of occurrence records as data from different databases are merged but not reconciled. In addition, therefore, occurrence data from aggregators suffers from inflation of the number of occurrences, as well as the poor and biased sampling effort identified above.

Similar inconsistencies arise with respect to spatial data because of transcription errors and modification of the data. For example, some contributors may use centroids to consist the data format, others may use label notes to generate spatial coordinates that were not recorded at the time of observation, and others may apply data to the wrong fields. For example, data entry undertaken at the Geneva herbarium (G) assigned latitude coordinates to longitude coordinates and vice versa; at the Instituto Nacional de Pesquisas da Amazônia (INPA), a single individual, ‘Mike Hopkins’ was listed in the ‘recordedBy’ field for every occurrence record, and at the Field Museum (F), centroid coordinates were applied. Once consolidated with other data sets, such errors and inconsistencies create superfluous occurrence records.

This can result in a single observation/data point being represented by thirty or more records (e.g. Gardner 417) with different species names and locality information. Such inflation leads to errors in species richness measures, both for higher taxon ranks but also for points on the globe, which in turn, can impact on ecological, biogeographic, and evolutionary studies^10–12^.

To mitigate duplication and errors in the global species occurrence dataset represented by GBIF, we aimed to harness two resources in an automated manner, the World Checklist of Vascular Plants (WCVP) taxonomic backbone^13^ and the fields appended to GBIF occurrence records. The WCVP taxonomic backbone is a database linking 1.4 million global plant names to 426,624 accepted species (https://wcvp.science.kew.org/). GBIF issues and flags (https://data-blog.gbif.org/post/issues-and-flags/) comprise > 50 appended fields, 33 of which correspond to geospatial issues.

We designed ParseGBIF to parse duplicate records into unique ‘collection events’ or ‘gatherings’, and in the process compile the most informative associated taxonomic and spatial data (where more than one duplicate is available). This required the generation of a unique identifier string combining the plant family name + first collector’s surname + the collection number and the generation of a collector dictionary, i.e. a list of the main collector names extracted from the dataset. We also used verbatim issues fields to score the completeness of each record with respect to geospatial and taxon identity fields, as well as to assess the consistency of the species epithet applied across duplicates of a unique collection event.

### Testing

ParseGBIF has been developed to work primarily with the GBIF database, which uses 100 different sources to assemble the GBIF backbone taxonomy^14^. We tested ParseGBIF using the records of the family Myrtaceae available from GBIF. This family was selected because of its relatively large size (ca 6000 species in 132 genera) and cosmopolitan distribution^15^. To evaluate the impact of ParseGBIF on species distribution records we asked the following questions: (1) How many duplicate records have been identified (and eliminated)? (2) How many unique collection events had duplicates with non-identical spatial data that was merged by ParseGBIF? (3) How many unique collection events with useable spatial data were recovered? (4) How was the average number of occurrence records per taxon impacted by the application of ParseGBIF? (5) Were there any differences between richness patterns of Myrtaceae produced by GBIF and ParseGBIF data?

## Results

We first describe a typical ParseGBIF workflow and then show the results of the workflow application to the distribution records of the family Myrtaceae.

### Typical ParseGBIF workflow

**Figure 1:**
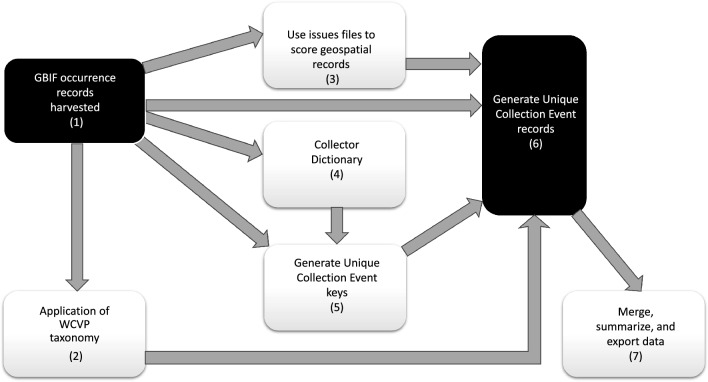
A typical ParseGBIF workflow.

ParseGBIF can be run as a single workflow, or as the individual functions to address specific tasks (Table 1, Fig. 1). ParseGBIF can also be run in association with other packages such as Gridder (Grid Detection and Evaluation in R, https://biogeographylab.github.io/GridDER.github.io/) or BDC^16^. After converting the GBIF data to a desirable format and selecting the most informative records, the resulting datasets can be downloaded and used in further analyses, e.g. spatial.

**Table 1:** A typical workflow for a ParseGBIF project.

Project step	Relevant function
GBIF data preparation	*download_gbif_data_from_doi* *prepare_gbif_occurrence_data* *select_gbif_fields* *extract_gbif_issue*
Check species names against WCVP database	*wcvp_get_data* *wcvp_check_name* *wcvp_check_name_batch* *standardize_scientificName*
Collectors Dictionary	*collectors_prepare_dictionary* *collectors_get_name* *generate_collection_event_key*
Selecting the master digital voucher	*select_digital_voucher*
Export dataset for further analysis	*export_data* *parseGBIF_summary*

#### GBIF data download and preparation

To download GBIF occurrence data, the DOI URL address generated by the manual search previously performed on the GBIF portal is entered as a parameter in the *download_gbif_data_from_doi* function. The function will download and unzip the file in the indicated destination folder. To prepare occurrence data downloaded from GBIF to be used by ParseGBIF functions, it is necessary to run *prepare_gbif_occurrence_data*. This will select the desired fields (“standard” or “all”) and rename them. The “standard” format has 54 data fields, and the “all” format, 257 data fields. The selection is enabled by the *select_gbif_fields* function.

As part of the data preparation, it is possible to check whether downloaded records have been identified by GBIF as having “issues”, i.e. either altered by GBIF during processing or flagged as potentially invalid, unlikely, or suspicious. From the list of all issues identified by GBIF (https://gbif.github.io/gbif-api/apidocs/org/gbif/api/vocabulary/OccurrenceIssue.html) we selected only those potentially influencing geospatial interpretation of records (33 categories, Supplementary Table S1). Each issue category was characterised as having a potentially no, low, medium or high impact on coordinate precision, and a selection score (0, − 1, − 3, − 9) were assigned to each record. The lowest selection score (-9) indicates records unsuitable for spatial analysis. The function *extract_gbif_issue* produces a summary showing the total number of records in each issue category.

#### Species name check against WCVP database

The World Checklist of Vascular Plants (WCVP) database (Royal Botanic Gardens Kew: https://powo.science.kew.org/about-wcvp) can be downloaded to a folder of the user's choice or into memory using get_wcvp function. The output has 33 columns.

Species’ names can be checked against the WCVP database one by one or in a batch mode. To verify individual names, the function *wcvp_check_name* is used. To check names in a batch mode, there is *wcvp_check_name_batch* function. It uses the occurrence data (“occ”) and WCVP names list (“wcvp_names”) generated in the previous steps.

To bring species’ names in line with the WCVP format, the function *standardize_scientificName* inserts space between the hybrid separator (x) and specific epithet, and also standardizes abbreviations of infrataxa (variety, subspecies, form). The function get_lastNameRecordedBy returns the surname of the main collector in “recordedBy” field.

#### Creating collectors dictionary

A necessary step for parsing duplicate records is to have a robust key for each unique collecting event (aka ‘gathering’). The inclusion of the plant family name in the collection event key, proposed by Nicolson^17^ avoids the merging of distinct collection events in the case of low collection numbers of common surnames. Including the collection event date in the key would be the most powerful way to avoid the merging of distinct collection events, however, collection date information is often not present in occurrence data records limiting the effectiveness of such an approach. We therefore generated a string that combined the plant family name + the first collector’s surname + the collection number^19^. For the event key to be effective it is essential to consistently record the collector surname and for this purpose we provide a collector dictionary, i.e. a curated list of the main collector surnames extracted from the GBIF dataset. *collectors_prepare_dictionary* function extracts the surname of the main collector, based on the “recordedBy” field and generates a list relating the surname of the main collector and the downloaded data. This is followed by the standardisation of the main collector’s last name in the “nameRecordedBy_Standard” field, with respect to lowercase and non-ascii characters. This ensures that a botanical collector is always recognized by the same surname. Where the searched name is present in the collector’s dictionary, then the function retrieves the surname of the main collector as recorded in the “recordedBy” field, at which point the “CollectorDictionary” field will be marked as ‘checked’. If not recognised, then the function returns the surname of the main collector, extracted automatically from the “recordedBy” field and the record will remain unchecked. Unchecked records are then reviewed manually. The Collector Dictionary supplied with ParseGbif contains all the collector surnames, checked, that were included in the GBIF download.

#### Digital voucher selection

Where duplicate records of unique collection events are present, the function *select_digital_voucher* selects the best record as the basis for the single unique collections event record. The best record is defined as that with the highest total score, for the sum of spatial data quality + record completeness.

Spatial data is scored based on the following GBIF issues (see Section "Typical ParseGBIF workflow" above):

- Not applicable, “selection_score” equals “zero”
- Does not affect coordinate accuracy, “selection_score” equals − 1
- Potentially affect coordinate accuracy, “selection_score” equals − 3
- Records to be excluded from spatial analysis, “selection_score” equals − 9

Record completeness scored is measured using the sum of the following flags being equal to TRUE (all “selection_score” equals 1):

- Is there information about the collector?
- Is there information about the collection number? Is there information about the year of collection?
- Is there information about the institution code?
- Is there information about the catalogue number?
- Is there information about the locality?
- Is there information about the municipality of collection?
- Is there information about the state/province of collection?
- Is there information about the country (using a GBIF issue COUNTRY_INVALID)?
- Is there information about the field notes?

The accepted TAXON_NAME selected is that which has most frequently been applied to the duplicate records at, or below, the rank of species. Where two names are applied with equal frequency, the first TAXON_NAME listed in alphabetical order is chosen to enable automation of the process. Where there is no identification at or below the rank of species, then the unique collection event is indicated as unidentified.

Where unique collection event duplicates cannot be parsed because the collection event key is incomplete, each record is treated as a unique collection event lacking duplicates. Where the collection event key is incomplete because the collector surname could not be recovered, then the record is tagged with the label, UNKNOWN-COLLECTOR.

#### Merged records

Once a master record is selected for each unique collection event key, duplicates can be removed from the dataset. For each complete unique collection event key, data fields that are empty in the digital voucher record will be populated with data from the respective duplicates. During content merging, we indicate fields associated with the description, location, and data of the unique collection event.

#### Outputs

Records can be exported at three levels of detail:

1. *All data* all records processed and merged, plus all records removed, either because they are duplicates of a unique collection event, or unusable. Records can be separated into three datasets (”useable”, “unusable” and “duplicates”) by filtering “parseGBIF_dataset_result” field.
2. *Usable records* unique collection events complete with WCVP taxonomic identification and useable spatial data. Duplicate and unusable records are excluded.
3. *Unusable records* unique collection events which lack key taxonomic and/or spatial data. This represents data that does not support taxonomic or spatial analyses but likely includes records that could be made useable through further manual checks.
4. *Duplicate records of unique collection events* duplicate records of the useable data could be useful for quality control or for verifying outlier records using a manual check.

Summary views of each export are available in the following configuration through the *parseGBIF_summary* function. The function summarizes the results of the *select_digital_voucher* (“occ_digital_voucher”) and *export_data* (“all_data”) functions. *parseGBIF_summary* function provides a general summary with totals for the entire dataset: total number of records/total number of unique collection events/total number of duplicate records of unique collection events/total number of usable records. The function *export_data*, with all records merged, provides a summary of the merged fields, summary of the merged fields for useable records only, including frequency of merge actions on fields in the usable dataset, and a summary of the unusable records, including the frequency of merge actions on fields in the unusable dataset.

### Example application

All available Myrtaceae records were download from GBIF (10.15468/dl.ykrqqv, May 2, 2023). The parameters of the download were: (1) Basis of record: preserved specimen; (2) Occurrence status: present; (3) Scientific name: Myrtaceae. In total, the GBIF download held information on 13,147 unique taxa; of which 9669 taxa had geographic coordinates suitable for spatial analysis (Table 2, Supplementary Fig. S1).

**Table 2:** GBIF and ParseGBIF datasets.

Dataset	All data			Data suitable for spatial analysis		
	Records	Number of taxa	Records/Taxon	Records	Number of taxa	Records/Taxon
GBIF	1,301,480	13,147	98.9	840,937	9669	86.9
ParseGBIF	1,301,480	6513	199.8	930,616	5964	156.0

#### Records modified (merged) by ParseGBIF workflow

Using ParseGBIF, 1,301,480 records representing 13,147 unique taxa downloaded from GBIF were parsed into 930,616 unique collection event records representing 5964 unique taxa (or 61.7% of the originally downloaded records). The parsing of duplicate records resulted in a doubling of the average number of records per taxa, from 98.9 to 199.8, where all records were parsed. Where only records suitable for spatial analysis were included, the parsing of duplicates resulted in a 1.8-fold increase, from 86.9 to 156. Of the unique collection events recovered, 252,723 (27%) had duplicates with non-identical spatial data.

**Figure 2:**
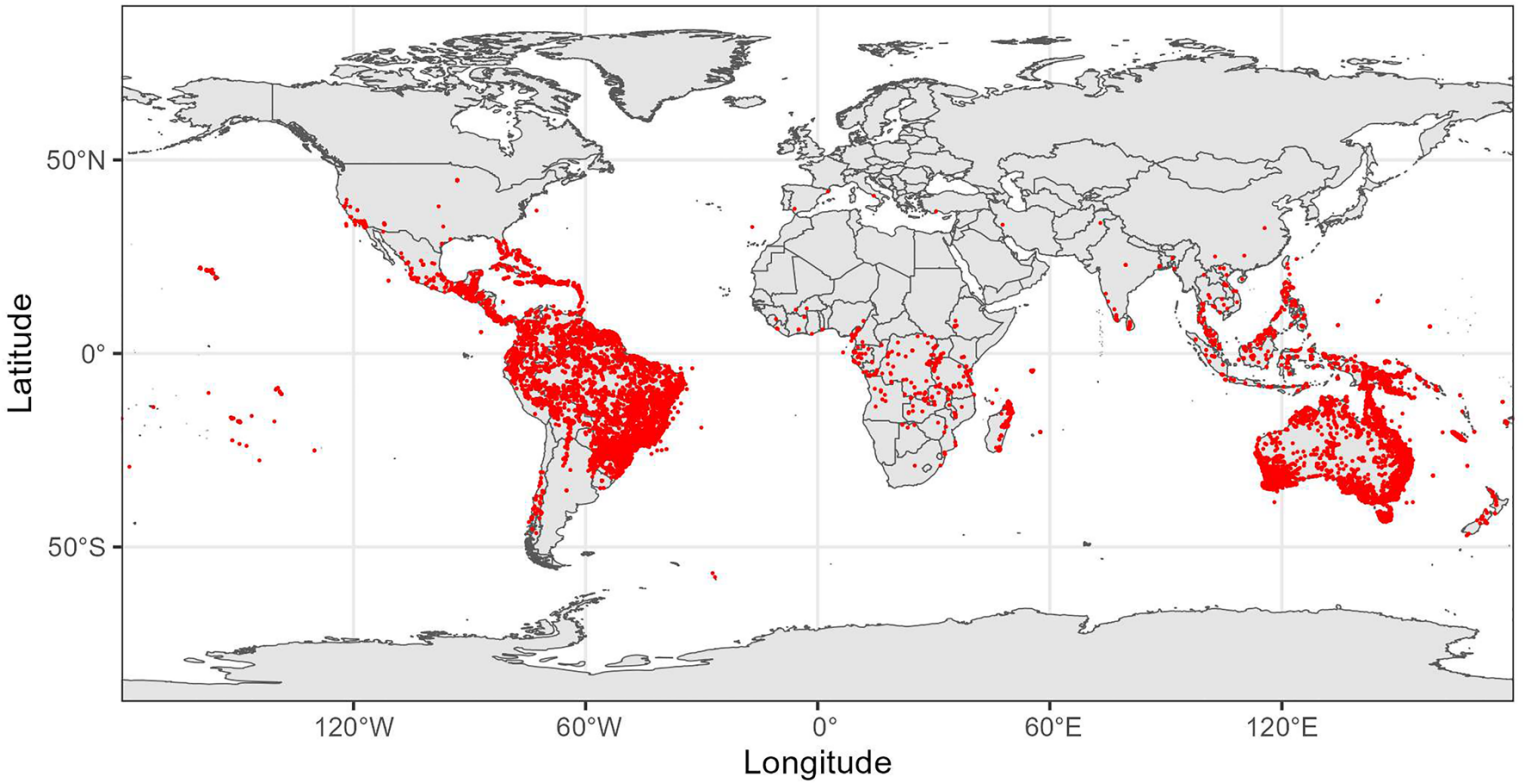
Modified (merged) Myrtaceae records (red dots). The highest density of the merged records overlaps with the areas of the high taxon richness (orange and red, see Fig. 3). Map created with custom R script. *Base map source* ESRI (http://www.esri.com/data/basemaps, © Esri, DeLorme Publishing Company).

In total, 37,156 records representing 3874 taxa modified by the ParseGBIF workflow; of those, 92% (3546 taxa) were suitable for spatial analysis (Fig. 2). The largest number of modifications to the occurrence related to the ‘Habitat’ field, followed by ‘Field Notes’ and ‘Municipality’ fields (Supplementary Table S2). Taxon richness, calculated for the parsed (merged) records was positively and significantly correlated with the overall taxon richness calculated for the ParseGBIF output (Pearson's product-moment correlation 0.6475, t = 40.5, df = 2271, *p*-value < 0.0001). This suggests that the areas of high taxonomic richness of Myrtaceae overlapped with the areas where the density of modified records was also high.

#### Myrtaceae taxon richness

**Figure 3:**
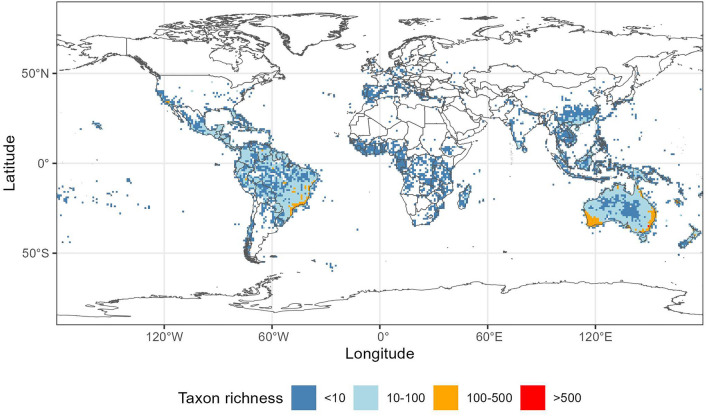
Myrtaceae taxon richness in 1 × 1 degree grid squares, ParseGBIF data. Map created with custom R script. *Base map source* ESRI (http://www.esri.com/data/basemaps, © Esri, DeLorme Publishing Company).

**Figure 4:**
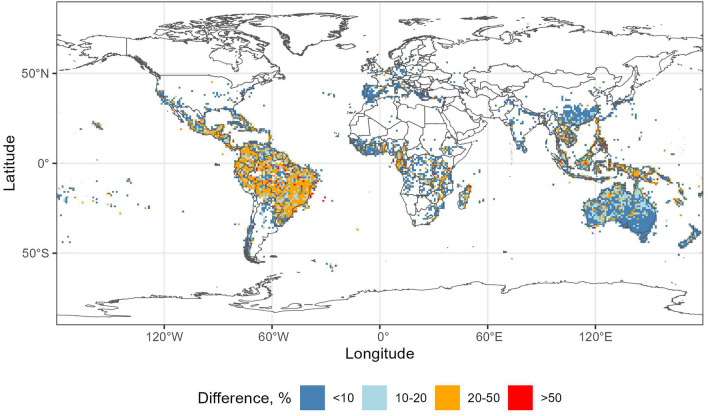
Percent difference between downloaded GBIF and ParseGBIF taxon richness of Myrtaceae in 1 × 1 degree grid squares. Map created with custom R script. *Base map source* ESRI (http://www.esri.com/data/basemaps,© Esri, DeLorme Publishing Company).

According to both GBIF and ParseGBIF data, the taxon diversity of Myrtaceae was, in general below, 100 taxa per 1 × 1 degree grid square (ca 110 × 110 km at the equator, Supplementary Fig. S1, Fig. 3). The highest taxon richness was found in the coastal areas of Brazil, and in Australia (Supplementary Fig. S1, Fig. 3). The largest difference between the downloaded GBIF and ParseGBIF taxon richness was found in Brazil (up to 50%, Fig. 4). However, the difference was generally rather small (below 20%) when expressed in taxon numbers (Supplementary Fig. S3). Raw GBIF and ParseGBIF taxon counts in 1 × 1 degree squares were positively and significantly correlated (Pearson's product-moment correlation 0.9699, t = 276.85, df = 4830, *p*-value < 2.2e−16).

#### Collector dictionary

The Myrtaceae dataset included 155,192 unique ‘recordedBy’ names, of which, 8.28% (12,855) were manually reviewed. The remaining names were suitable for automated processing. The unique collection event with the most duplicates was MYRTACEAE_GARDNER_417, which had 32 duplicates in 13 herbaria (E, K, NHMUK, UEFS, S, F, US, CAS, NY, A, GH, W and MNHN (*Campomanesia hirsuta* Gardner).

## Discussion

ParseGBIF offers a significant improvement on using records as downloaded from GBIF, greatly increasing the number of occurrence records per taxon. This was achieved mainly through the consisting of taxon names, the consisting of spatial data, and the merging of duplicate records, both of which mitigate the inflation in records identified above (See Introduction). ParseGBIF workflow did, not, however, alter the species richness patterns for the Myrtaceae dataset. For studies solely interested in species patterns at the global or regional scale, GBIF occurrence records as available through the GBIF download function do not need further processing. For finer scale analyses of occurrence data, however, ParseGBIF will add significant value. For example, for the evaluation of species’ occurrences at the national scale, in support of national biodiversity plans, or plant areas important for biodiversity, or the characterisation of sample bias to inform future sampling effort, or the use of occurrence data to model species’ niches, and or in support of species Red Listing.

### Comparison of ParseGBIF to other packages

According to a recent review^18^ there are at least 17 packages that offer taxonomic record cleaning functions for plants (https://mgrenie.shinyapps.io/taxharmonizexplorer/). ParseGBIF is unique in the recovery of unique collection events from GBIF occurrence records, through the application of a collector dictionary and the selection of ranked spatial data. Three other packages (kewr, rWCVP, and treemendous) also apply the WCVP taxonomic backbone. We feel that the harnessing of issues with occurrence records, flagged by GBIF, to score the spatial data and rank duplicate records provides a novel way to resolve conflicts in spatial data between duplicate records.

The ParseGBIF workflow is most similar to that developed by CNCFlora^19^ for the curation of distribution records. ParseGBIF differs from Moura et al.^19^ workflow with respect to the method for obtaining occurrence records; ParseGBIF uses a URL generated by a download request on the GBIF portal, whereas the former uses a predetermined list of scientific names. This enables ParseGBIF to process records by taxonomic group or spatial area. In addition, ParseGBIF consists the taxon name applied to duplicates records in an automated manner and aligns them with the WCVP, whilst Moura et al.^19^ workflow consists names manually and aligns them with the Flora do Brasil 2020^20^. Furthermore and as above, ParseGBIF uses GBIF issues to rank and select the optimum duplicate record spatial data from across the globe; in contrast, Moura et al.^19^, applies a more rigorous validation of geographic coordinates, cross-referencing the municipality and state together against the CNCFlora gazetteer, but this is restricted to Brazil. Finally, ParseGBIF populates empty unique collection event record data fields with ranked data, where present, from the respective duplicate records, enabling a complete record to be assembled, even where none of the duplicate records are complete. Such a function is not present in Moura et al^19^.

Elements of ParseGBIF are similar to a clustering function recently introduced by GBIF, which allows users to identify records that appear to be derived from the same source (https://www.gbif.org/news/4U1dz8LygQvqIywiRIRpAU/new-data-clustering-feature-aims-to-improve-data-quality-and-reveal-cross-dataset-connections; https://data-blog.gbif.org/post/clustering-occurrences/). GBIF, however, does not use this function to parse the duplicate records into unique collection events, but rather to estimate the extent of duplication within a dataset. In contrast, ParseGBIF removes the duplicates.

We feel that the Collector Dictionary, a list of the main collector names extracted from the dataset, is a useful and important resource that can also be used independently of the ParseGBIF workflow. We see scope for the strengthening the ParseGBIF workflow by adding more active ‘cleaning’ of the spatial data, through the integration of other work packages such as Gridder (Grid Detection and Evaluation in R, https://biogeographylab.github.io/GridDER.github.io/), bdc (A toolkit for standardizing, integrating and cleaning biodiversity data, https://brunobrr.github.io/bdc/), and functions from the CoordinateCleaner package, such as Automated Cleaning of Occurrence Records from Biological Collections (https://cran.r-project.org/package=CoordinateCleaner). The Collector Dictionary differs from similar initiatives such as Bionomia (https://bionomial.net) in that it uses the raw collector names information ("recordedBy" field) from GBIF as the primary key, enabling teams of collectors from different data sources to be indexed. In addition, the Collector Dictionary standardized surname does not necessarily represent a unique individual as is the case in Bionomia.

### The impact of ParseGBIF on species distribution records

When applied to the Myrtaceae dataset, ParseGBIF had a marked impact on the number of occurrence records and taxon names: over half of taxon names were eliminated, bringing estimated species-richness of the Myrtaceae close to that estimated in the WCVP^21^. In response, the average number of occurrence records with usable spatial data, per taxa, nearly doubled making them more suitable for statistical and/or spatial modelling. The removal of duplicate records and those lacking the spatial quality for spatial analysis, reduced the number of taxa from 6513 to 5964. A visual comparison of species richness patterns before (Supplementary Fig. S1) and after (Fig. 3) applying the ParseGBIF workflow clearly illustrates the above: a straight line in the South Atlantic representing records with erroneous coordinates (equal longitude and latitude coordinates, Supplementary Fig. S1) being absent from the processed records (Fig. 3). Not all geospatial issues, however, could be resolved by ParseGBIF, including country and province centroids and records “falling” off the coast, i.e. located beyond the coastline due to georeferencing errors. For this reason, we advise the application of ParseGBIF in combination with Gridder or bdc.

Of the unique collection events recognised, nearly 60% are suitable for spatial analysis and were included in the modified (merged) fields. The merged fields had a higher information content, particularly with respect to habitat and collection locality descriptions, important information in high resolution ecological and niche studies. The strong correlation between species richness in the modified cells, compared to unmodified cells suggests that ParseGBIF workflow does not impact the mapping of species distributions at the global or regional scale, but that it could provide valuable information at the fine (national subnational) scale and in species-poor, potentially under-sampled locations.

### The impact of ParseGBIF on taxon richness patterns of Myrtaceae

The patterns of taxon richness produced by GBIF and ParseGBIF records were similar and consistent with the earlier assessments of Myrtaceae distribution^15^. Brazil and Australia appeared to be centres of taxon richness with over 100 taxa per 1 × 1 degree grid square. Two notable exceptions were Borneo and Thailand where taxon richness was lower than expected^15^.

When compared, per grid square taxon richness of Myrtaceae estimated using GBIF and ParseGBIF data differed by 20 taxa or less in the majority of grid squares. There was a strong positive significant correlation (r = 0.97) between taxon richness produced by GBIF and ParseGBIF records suggesting that the workflow did not alter taxon richness distribution patterns at the global scale.

### Supplementary Information

Supplementary Information.

## Data Availability

The ParseGBIF R package and Manual are available from GitHub: https://github.com/pablopains/parseGBIF. The Myrtaceae dataset is available from Figshare: Bystriakova, Nadia; de Melo, Pablo Hendrigo Alves; Monro, Alexandre (2023). Myrtaceae Dataset. Figshare. Dataset. https://doi.org/10.6084/m9.figshare.24465733.v1.
