# Supplementary material for: A new R package to parse plant species occurrence records into unique collection events efficiently reduces data redundancy
Source: Sci Rep. 2024 Mar 5;14:5450. doi: 10.1038/s41598-024-56158-3 (PMC10914741; doi:10.1038/s41598-024-56158-3)
Supplement: Supplementary file 1 — Supplementary Information. [file 41598_2024_56158_MOESM1_ESM.pdf]

Supplementary Information

A new R package to parse plant species occurrence records into unique collection events efficiently reduces data redundancy

Pablo Hendrigo Alves de Melo, Nadia Bystriakova, Eve Lucas, Alexandre Monro

Table S1. Interpretation of GBIF geospatial issues (https://data-blog.gbif.org/post/issues-and-flags/).

	GBIF geospatial issue	Description	Priority	Selection score	Reasoning
**1**	COORDINATE_UNCERTAINTY_METERS_INVALID	Indicates an invalid or very unlikely dwc:uncertaintyInMeters.	Low	-1	Does not affect coordinate accuracy
**2**	CONTINENT_COORDINATE_MISMATCH	The interpreted occurrence coordinates fall outside of the indicated continent.	Low	-1	Does not affect coordinate accuracy
**3**	CONTINENT_COUNTRY_MISMATCH	The interpreted continent and country do not match.	Low	-1	Does not affect coordinate accuracy
**4**	CONTINENT_DERIVED_FROM_COORDINATES	The interpreted continent is based on the coordinates, not the verbatim string information.	Low	-1	Does not affect coordinate accuracy
**5**	CONTINENT_DERIVED_FROM_COUNTRY	The interpreted continent is based on the country, not the verbatim string information.	Low	-1	Does not affect coordinate accuracy

	GBIF geospatial issue	Description	Priority	Selection score	Reasoning
**6**	COORDINATE_ACCURACY_INVALID	Deprecated.B	Low	-1	Does not affect coordinate accuracy
**7**	COORDINATE_PRECISION_INVALID	Indicates an invalid or very unlikely coordinatePrecision	Low	-1	Does not affect coordinate accuracy
**8**	COORDINATE_PRECISION_UNCERTAINTY_MISMATCH	Deprecated.B	Low	-1	Does not affect coordinate accuracy
**9**	COORDINATE_REPROJECTED	The original coordinate was successfully reprojected from a different geodetic datum to WGS84.	Low	-1	Does not affect coordinate accuracy
**10**	ELEVATION_NON_NUMERIC	Set if elevation is a non-numeric value	Low	-1	Does not affect coordinate accuracy
**11**	ELEVATION_NOT_METRIC	Set if supplied elevation is not given in the metric system, for example using feet instead of meters	Low	-1	Does not affect coordinate accuracy
**12**	ELEVATION_UNLIKELY	Set if elevation is above the troposphere (17km) or below 11km (Mariana Trench).	Low	-1	Does not affect coordinate accuracy
**13**	CONTINENT_INVALID	Uninterpretable continent values found.	Low	-1	Does not affect coordinate accuracy
**14**	COUNTRY_DERIVED_FROM_COORDINATES	The interpreted country is based on the coordinates, not the verbatim string information.	Low	-1	Does not affect coordinate accuracy

	GBIF geospatial issue	Description	Priority	Selection score	Reasoning
**15**	COUNTRY_INVALID	Uninterpretable country values found.	Low	-1	Does not affect coordinate accuracy
**16**	ELEVATION_MIN_MAX_SWAPPED	Set if supplied minimum elevation > maximum elevation	Low	-1	Does not affect coordinate accuracy
**17**	COORDINATE_ROUNDED	Original coordinate modified by rounding to 5 decimals.	Low	-1	Does not affect coordinate accuracy at 1x1 km or coarser resolution
**18**	DEPTH_MIN_MAX_SWAPPED	Set if supplied minimum depth > maximum depth	None	0	Not applicable
**19**	DEPTH_NON_NUMERIC	Set if depth is a non-numeric value	None	0	Not applicable
**20**	DEPTH_NOT_METRIC	Set if supplied depth is not given in the metric system, for example using feet instead of meters	None	0	Not applicable
**21**	DEPTH_UNLIKELY	Set if depth is larger than 11,000m or negative.	None	0	Not applicable
**22**	COUNTRY_MISMATCH	Interpreted country for dwc:country and dwc:countryCode contradict each other.	Low	-1	Possibly due to geopolitical changes. Does not affect coordinate accuracy.
**23**	COORDINATE_REPROJECTION_FAILED	The given decimal latitude and longitude could not be reprojected to WGS84 based on the provided datum.	Medium	-3	Potentially affect coordinate accuracy

	GBIF geospatial issue	Description	Priority	Selection score	Reasoning
**24**	COORDINATE_REPROJECTION_SUSPICIOUS	Indicates successful coordinate reprojection according to provided datum, but which results in a datum shift larger than 0.1 decimal degrees.	Medium	-3	Potentially affect coordinate accuracy
**25**	GEODETIC_DATUM_INVALID	The geodetic datum given could not be interpreted.	Medium	-3	Potentially affect coordinate accuracy
**26**	PRESUMED_NEGATED_LATITUDE	Latitude appears to be negated, e.g.	Medium	-3	Potentially affect coordinate accuracy
**27**	PRESUMED_NEGATED_LONGITUDE	Longitude appears to be negated, e.g.	Medium	-3	Potentially affect coordinate accuracy
**28**	PRESUMED_SWAPPED_COORDINATE	Latitude and longitude appear to be swapped.	Medium	-3	Potentially affect coordinate accuracy
**29**	GEODETIC_DATUM_ASSUMED_WGS84	Indicating that the interpreted coordinates assume they are based on WGS84 datum as the datum was either not indicated or interpretable.	Medium	-3	Potentially affect coordinate accuracy, if datum is not WGS84
**30**	COORDINATE_INVALID	Coordinate value is given in some form but GBIF is unable to interpret it.	High	-9	Records to be excluded from spatial analysis
**31**	COORDINATE_OUT_OF_RANGE	Coordinate has a latitude and/or longitude value beyond the maximum (or minimum) decimal value.	High	-9	Records to be excluded from spatial analysis

	GBIF geospatial issue	Description	Priority	Selection score	Reasoning
**32**	COUNTRY_COORDINATE_MISMATCH	The interpreted occurrence coordinates fall outside of the indicated country.	High	-9	Records to be excluded from spatial analysis
**33**	ZERO_COORDINATE	Coordinate is the exact 0B°, 0B° coordinate, often indicating a bad null coordinate.	High	-9	Records to be excluded from spatial analysis

Table S2. Summary of merged fields. DWC definition is provided according to Darwin Core List of Terms (http://rs.tdwg.org/dwc/doc/list/2023-09-18).

Merged field	Number of records	DWC definition
Ctrl_habitat	16903	A category of description of the habitat in which the dwc:Event occurred.
Ctrl_fieldNotes	9079	Either, a) an indicator of the existence of, b) a reference to (publication, URI), or c) the text of notes taken in the field about the dwc:Event.
Ctrl_municipality	8583	The full, unabbreviated name of the next smaller administrative region than county (city, municipality, etc.) in which the dcterms:Location occurs.
Ctrl_stateProvince	3884	The name of the subsequent administrative region below country in which dcterms:Location occurs.
Ctrl_year	3639	The four-digit year in which the dwc:Event occurred, according to the Common Era Calendar.
Ctrl_eventDate	3639	The date-time or interval during which a dwc:Event occurred. For occurrences, this is the date-time when the dwc:Event was recorded. Not suitable for a time in a geological context.

Merged field	Number of records	DWC definition
Ctrl_locality	2344	Less specific geographic information can be provided in other geographic terms (dwc:higherGeography, dwc:continent, dwc:country, dwc:stateProvince, dwc:county, dwc:municipality, dwc:waterBody, dwc:island, dwc:islandGroup). This term may contain information modified from the original to correct perceived errors or standardize the description.
Ctrl_level0Name	1249	Additional properties from database of Global Administrative Areas level 0 subdivision or Country. Is the familiar concept of first-level political entity
Ctrl_level1Name	1250	Additional properties from database of Global Administrative Areas level 1 subdivision or State/Province. Is a primary subdivision of a country, be it state, province, department.
Ctrl_level2Name	1242	Additional properties from database of Global Administrative Areas level 2 subdivision or County. Is a

Merged field	Number of records	DWC definition
		second-level political subdivision of a country, regardless of local labels.
Ctrl_level3Name	302	Additional properties from database of Global Administrative Areas level 3 subdivision. Is a third-level political subdivision of a country, regardless of local labels.
Ctrl_countryCode	184	The standard code for the country in which the dcterms:Location occurs.

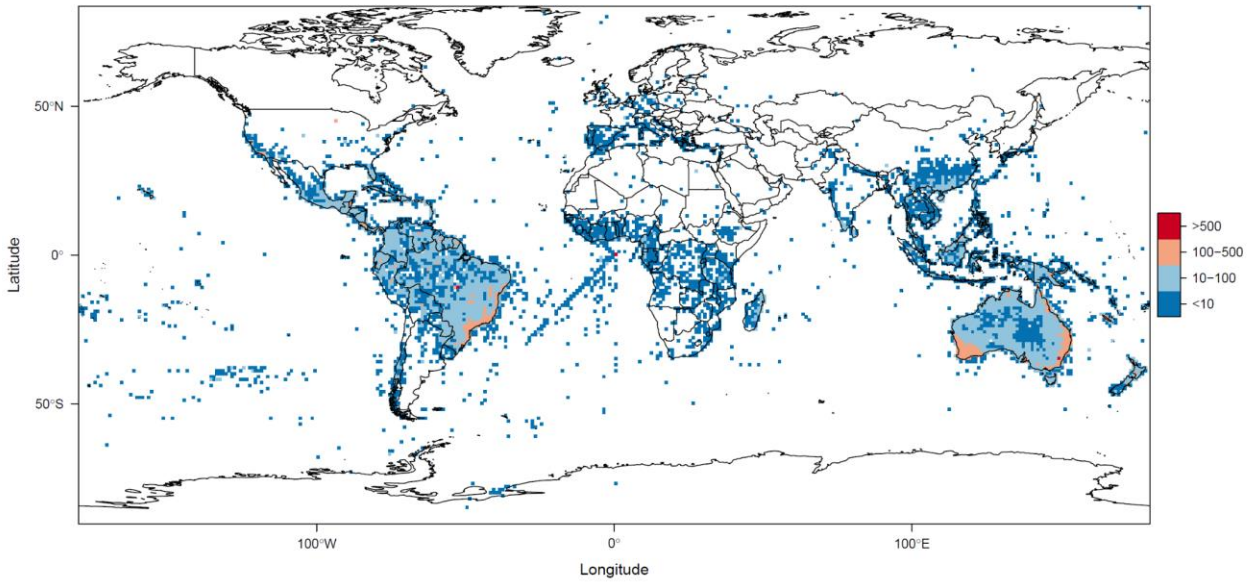

Figure S1. Myrtaceae taxon richness in 1 x 1 degree grid squares, GBIF data. A geometric pattern produced by records with zero-zero and longitude-equal-latitude coordinates is visible near the western coast of the African continent. Map created with custom R script. Base map source: ESRI (http://www.esri.com/data/basemaps, © Esri, DeLorme Publishing Company).

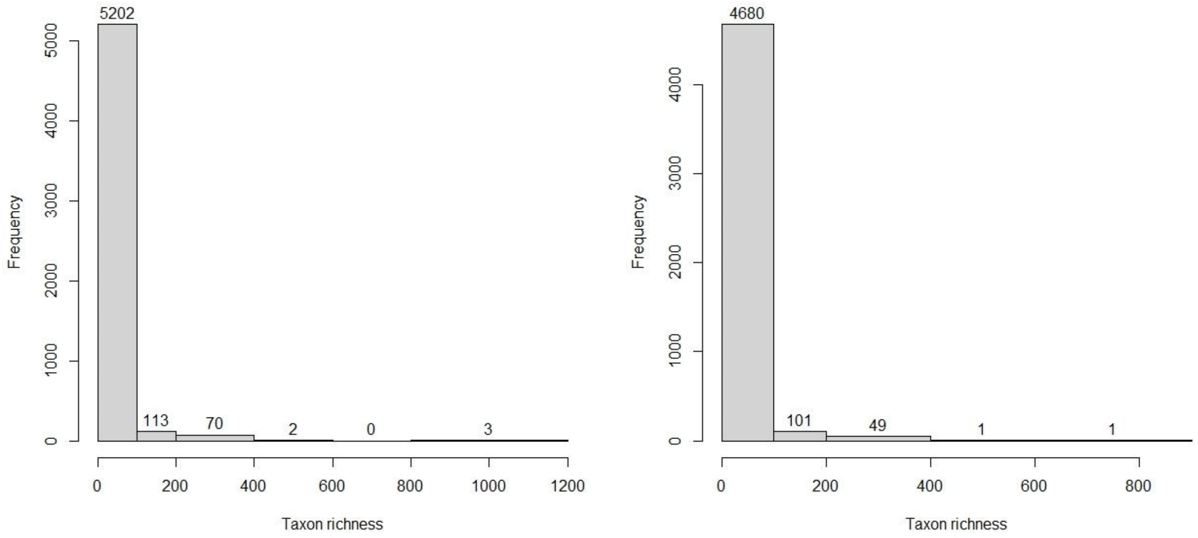

Figure S2. Myrtaceae taxon richness in 1 x 1 degree grid squares, GBIF (left) and ParseGBIF (right) data.

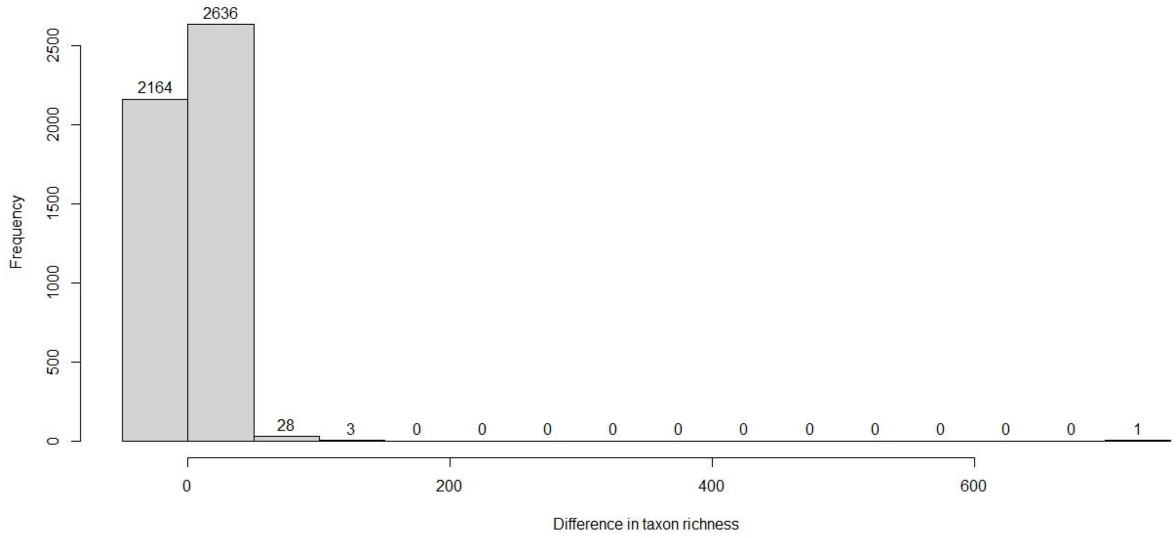

Figure S3. Difference between GBIF and ParseGBIF in taxon richness in 1 x 1 degree grid squares. The majority of values are below 20, with a single grid square (far right) having 736 taxa.
